# Scratching the surface – tobacco-induced bacterial biofilms

**DOI:** 10.1186/s12971-014-0026-3

**Published:** 2015-02-10

**Authors:** Justin A Hutcherson, David A Scott, Juhi Bagaitkar

**Affiliations:** Departments of Microbiology and Immunology, University of Louisville, Louisville, USA; Oral Immunology and Infectious Diseases, University of Louisville, 501 South Preston Street, Louisville, KY 40292 USA; Pediatrics, Washington University School of Medicine, Saint Louis, MO USA

**Keywords:** Bacteria, Biofilms, Cigarette smoking, Infectious diseases, Outer membrane, Tobacco

## Abstract

Individual environmental factors, such as iron, temperature and oxygen, are known to have a profound effect on bacterial phenotype. Therefore, it is surprising so little known is about the influence of chemically complex cigarette smoke on bacterial physiology. Recent evidence has demonstrated that tobacco smoke and components alter the bacterial surface and promote biofilm formation in several important human pathogens, including *Staphylococcus aureus, Streptococcus mutans, Klebsiella pneumonia, Porphyromonas gingivalis* and *Pseudomonas aeruginosa*. The mechanisms underlying this phenomenon and the relevance to increased susceptibility to infectious disease in smokers and to treatment are reviewed.

## Introduction

The numbers of smokers and, subsequently, tobacco-induced deaths continues to rise globally. In addition to cancers, chronic lung disease and cardiovascular complications, cigarette use is a major risk factor for multiple bacterial infections. These include biofilm-associated diseases, such as community-acquired pneumonia, otitis media, vaginosis and chronic periodontitis [1]. While there has been considerable focus on the mechanisms by which smoking dysregulates the immune system [2,3], little information is available as to how smoking influences the actual bacteria that cause disease. It is clear, however, that many bacteria exhibit a high degree of tolerance to cigarette smoke and smoke components [4-9], while it has been known for some considerable time that tobacco components can even promote the growth of some pathogenic bacteria, such as *Haemophilus influenzae* [10]. This review summarizes the available literature on tobacco smoke augmentation of biofilm formation by several important human pathogens. While it is apparent that we are only scratching the surface, mechanistically, potential explanations for smoke-induced biofilm enhancement are discussed.

## Methods

### Search strategy

Pubmed was investigated on 5 May 2014 and again on manuscript revision according to the following search strategies. [#1 (tobacco OR cigar* OR smok* OR nicotine) AND biofilm]; [#1 AND #2 (bacter*) AND coloniz*]; [#1 AND #2 AND membrane]; [#1 AND #2 AND ultrastructure]; [#1 AND #2 AND LPS]; [#1 AND #2 AND fimbria*]; [#1 AND #2 AND flagella]; [#1 AND #2 AND lipoteichoic]; [#1 AND #2 AND adhesion]; [#1 AND #2 AND adhesin]; and [#1 AND #2 AND peptidoglycan]. Data identified as generated by the tobacco industry was excluded *a priori*.

## Results and discussion

### Biofilm formation and bacterial survival

Biofilms are dense, surface-attached communities of bacteria or fungal species enclosed within a microbial-derived matrix that facilitates colonization and survival. The chemical composition, complexity and microbial diversity of a biofilm can vary relative to its environment. However the dynamics of biofilm formation can be generalized into three interlinked stages.

Attachment of planktonic bacteria to abiotic or biotic surfaces comprises the first stage of biofilm formation. Attachment occurs after detection of optimal nutrient or host-derived metabolite concentrations, pH, temperature and other favorable environmental signals [11]. Microbe-derived adhesive proteins and adhesive organelles, like fimbriae and pili, facilitate attachment of planktonic bacteria to the tissue or abiotic surface. The expression profiles of genes involved in bacterial motility, extracellular matrix formation, quorum sensing, chemotaxis and posttranscriptional regulatory circuits converge to promote colonization and establishment of biofilms [12,13]. Significant human pathogens, including *Pseudomonas aeruginosa, Vibrio cholerae* and *Escherichia coli*, all undergo extensive morphological and transcriptional changes to attach and colonize the host [12].

Following initial attachment, secondary species may bind to these early colonizers, increasing biofilm complexity. During the second stage of biofilm formation, cellular replication, synergistic intra- and inter-species interactions promote microcolony formation and the deposition of an extracellular matrix. Microcolonies grow as a direct consequence of bacterial replication and aggregation [14]. The extracellular matrix consists of exopolysaccharies, extracellular DNA, RNA, matrix-associated proteins and adhesins synthesized by the colonizing bacteria which promotes cell-to-cell adhesion and further stabilizes biofilm architecture. There may be several niches or microenvironments generated within a biofilm due, for example, to nutrient, oxygen and pH gradients. These gradients create selective pressure and can also enhance the pathogenic potential of an organism. The extracellular matrix also presents a diffusion barrier for most antimicrobial peptides and antimicrobial compounds.

Biofilms have been estimated to account for 65% or more of all microbial infections in humans [15]. This has critical implications to disease prevention as such microbial communities provide several advantages to the bacteria, such as (i) enhanced evolution through the sharing of genetic material, including antibiotic resistance and other virulence factor genes; (ii) protection from antibiotics; (iii) shielding from critical components of the immune response, including the complement/antibody system and phagocytosis; and (iv) the potential to further colonize the host upon shedding from the biofilm [15-18]. Given their high degree of resistance to current anti-microbial compounds, biofilms play a significant role in the pathogenesis of many chronic human infections, such as cystic fibrosis, bacterial endocarditis and periodontal diseases [19]. Furthermore, biofilms can prolong inflammation and delay resolution in chronic wound infections [20]. Several recent reviews of infectious biofilms are available [15,18,21-24].

### Tobacco smoke promotes biofilm formation

Simple environmental stimuli, including temperature, pH and the availability of iron, have a major influence on the bacterial transcriptome and influence biofilm formation in, for example, *Pseudomonas aeruginosa*, *Klebsiella pneumonia*, *Porphyromonas gingivalis*, and the melioidosis pathogen, *Burkholderia pseudomallei* [25-28]. It is perhaps not surprising, then, that cigarette smoke, which contains thousands of chemicals, can exert a profound influence on bacterial physiology and biofilm formation.

**Figure 1:**
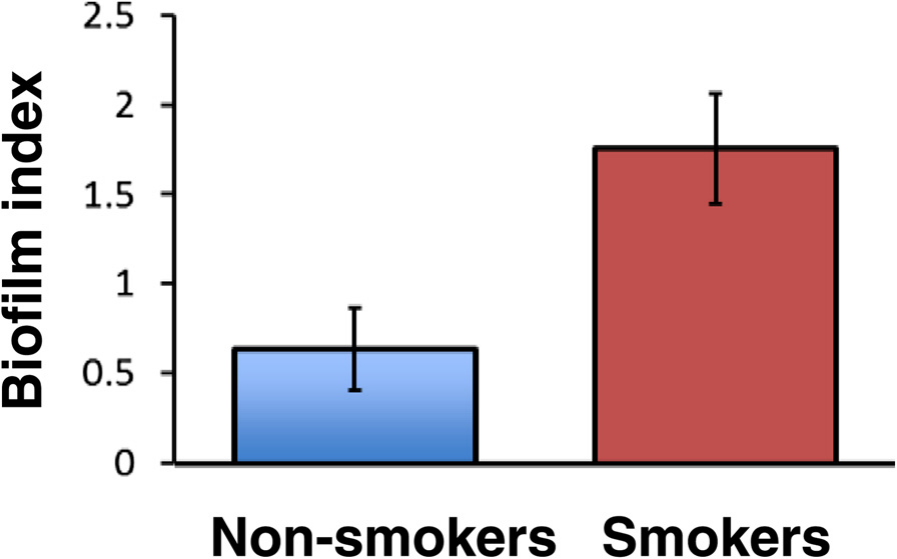
**Cigarette smoke enhances the biofilm index of multiple paranasal sinus isolates** ***.*** Eighteen pathogenic bacterial strains were isolated from non-smokers and 16 from smokers with sinonasal mucopurulence and evaluated for biofilm forming capacity after three hour tobacco smoke exposure from five 1R5F reference cigarettes or sham exposure. Data from all strains was then normalized by creating a ratio of smoke to sham exposed biofilm formation. A value of <1 demonstrates biofilm inhibition while a value of >1 reflects biofilm induction. The biofilm index was significantly different between the isolates from smokers and non-smokers (*p* < 0.001). Data is taken from Goldstein-Daruech et al. [29].

The evidence that tobacco promotes bacterial biofilms in multiple pathogenic bacteria is growing, as summarized in Table 1 along with their associated diseases. The most extensive study to date is by Goldstein-Daruech et al. [29], who demonstrated that acute cigarette smoke exposure significantly increased biofilm formation in 75% (12/16) of clinical isolates of various species from smokers with chronic rhinosinusitis but in 0% (0 of 18) of isolates from nonsmokers. Importantly, such enhanced biofilm formation was reversible on removal of the cigarette smoke stimulus. An overall biofilm index for smokers and non-smokers is reproduced from the Goldstein-Daruech study in Figure 1.

**Table 1:** **Tobacco augments biofilm formation in multiple human pathogens**

**Bacterium** **(Gram)**	**Disease**	**Stimulus** ^**1**^	**Study**
*Klebsiella pneumonia* (−)	Chronic rhinosinusitis isolate	Whole smoke	Goldstein-Daruech et al. 2011 [29]
*Pseudomonas aeruginosa* (−)	Nosocomial infections; UTI; respiratory infections including pneumonia	Whole smoke	Antunes 2012 [30]
			Goldstein-Daruech et al. 2011 [29]
*Porphyromonas gingivalis* (−)	Chronic periodontitis	CSE	Bagaitkar et al. 2009, 2011 [1,31]^2^
*Proteus vulgaris* (−)	Chronic rhinosinusitis isolate	Whole smoke	Goldstein-Daruech et al. 2011 [29]
*Staphylococcus aureus* (+)	Nosocomial infections; endocarditis; osteomyelitis; respiratory infections	CSE	Kulkarni 2013 [32]
			Goldstein-Daruech et al. 2011 [29]
*Streptococcus pneumoniae* (+)	Pneumonia, bronchitis; endocarditis; meningitis	CSC	Cockeran et al. 2014 [33]
			Mutepe et al. 2012 [34]
			Goldstein-Daruech et al. 2011 [29]
*Streptococcus mutans* (+)	Dental caries	Nicotine, CSC	Li 2014 [8]^3^
			Huang et al. 2012 [35]
			Baboni 2010 [36]

^1^See references for precise methods of whole smoke exposure, CSE (cigarette smoke extract) or CSC (cigarette smoke condensate) preparation and of biofilm quantification.

Dual species biofilms with *S. gordonii* ^2^ or *S. sanguinis* ^3^.

### Mechanisms of tobacco smoke-enhanced biofilm formation

We are only beginning to understand how tobacco smoke may enhance microbial biofilms. However, it is clear that the first step in biofilm formation is adherence to a stratum, be that epithelial or endothelial cells; extant colonized bacteria; or to a sugar or protein. Tobacco smoke augments binding of *Streptococcus pneumonia* to pulmonary epithelial cells by inducing eukaryotic platelet-activating factor receptor (PAF-R) expression, which interacts with phosphorylcholine on the bacterial cell wall [37]. In *P. gingivalis*, the major fimbrial protein, FimA, is upregulated, which aids adhesion by binding to the glyceraldehyde 3-phosphate dehydrogenase (GAPDH) surface protein of the primary periodontal colonizer, *Streptococcus gordonii* [31]. The predominant nicotine metabolite, cotinine, has been reported to increase *P. gingivalis* adhesion to epithelial cell monolayers [4]. Cigarette smoke extract (CSE) also appears to promote adhesion of *Aggregatibacter actinomycetemcomitans*, an oral biofilm dweller associated with a localized, aggressive form of periodontal disease, to epithelial cells [38].

Sortase A (cell surface protein P1) is employed by the cariogenic agent, *Streptococcus mutans*, and other Gram-positive bacteria, to facilitate the localization of specific, LPXTGX-containing proteins to the microbial surface. One such protein is the salivary agglutinin-binding, biofilm promoting antigen I/II. It has recently been shown that the tobacco alkaloid, nicotine, upregulates the surface expression of antigen I/II by *S. mutans* and, subsequently, enhances biofilm formation [9]. The authors [9] suggest that this nicotine-enhanced biofilm formation helps explain the increased number of teeth with carious lesions found in smokers compared to non-smokers [39]. Indeed, nicotine has also been shown to enhance dual species *S. mutans* biofilm formation with *Streptococcus sanguinis* [8].

Cigarette smoke exposure also leads to increased biofilm formation in the key human pathogen, *Staphylococcus aureus*, which can cause, for example, skin infections, pneumonia, endocarditis, and septic shock [32]. The staphylococcal genes, accessory regulator A (*sarA*) and required for biofilm formation (*rbf*)*,* which encode biofilm enhancing proteins, and fibronectin binding protein A (*fnbA*), whose gene product facilitates bacterial adhesion, are upregulated by cigarette smoke. The accessory gene regulator (*agr)* family of genes is involved in quorum sensing which controls bacterial dispersal. *agrC* activity is suppressed by cigarette smoke. Cigarette smoke-enhanced biofilm formation in *S. aureus* is abrogated by pre-treatment with the antioxidant, *N*-acetyl cysteine, suggesting that smoke-induced *S. aureus* biofilm formation is oxidant-dependent [32].

Whole cigarette smoke exposure has been reported to increase biofilm formation in other pathogens but, again, there is little mechanistic insight. Several biofilm promoting *P. aeruginosa* genes (*pilF*, *flgK*) are induced by cigarette smoke, while the quorum sensing gene, *rhlA*, is suppressed [30]. Cigarette smoke condensate has also been shown to increase *Streptococcus pneumoniae* biofilm formation associated with a reduced production of pneumolysin, a key mediator of *S. pneumoniae*-induced inflammation [34].

### Tobacco smoke and oral biofilms

Presumably due to ubiquity of dental plaque, the high prevalence of bacteria-induced oral diseases, and ease of access to clinical samples, our knowledge of the influence of smoking on biofilms is broadest for those found in periodontal tissues. Oral biofilms are complex and colonize both supra- and sub-gingival regions of the oral cavity and their composition can correlate with increased severity of periodontal disease [40].

Chronic periodontitis is a tobacco-induced and/or exacerbated disease [41]. Multiple studies have established that smoking alters the bacterial composition of dental plaque, with several important periodontal pathogens – including *Treponema denticola*, *Fusobacterium nucleatum* and *P. gingivalis*, over-represented in cigarette users, relative to non-smokers [1,41-45]. Commensal species, such as *Streptococcus* species may be in higher abundance in non-smokers compared to smokers [44]. Interestingly, smokers who quit show a reversion to a healthier biofilm composition after 6 months [46].

**Figure 2:**
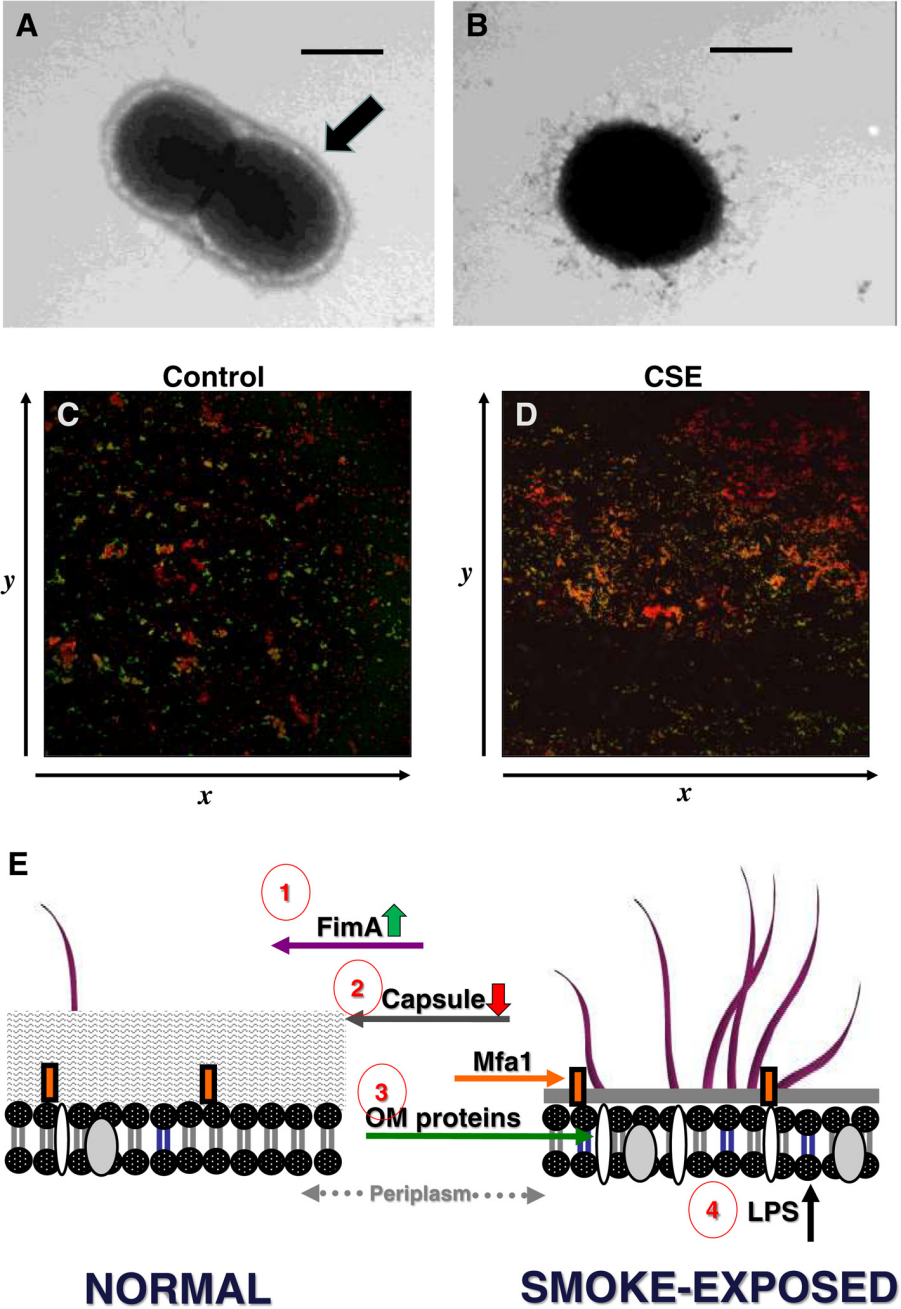
**Cigarette smoke extract alters key** ***P. gingivalis*** **surface molecules and enhances biofilm formation.** Representative transmission electron microscope images of *P. gingivalis* grown in control medium **(A)** or CSE-conditioned medium **(B)** are shown. The black arrow indicates the *P. gingivalis* capsule, which is greatly reduced in the presence of CSE. Images and data taken from Bagaitkar et al. [6]. **(C, D)** *S. gordonii* cells (hexidium iodide-labeled, red) were placed on a saliva-coated coverglass in a flow cell. *P. gingivalis* cells (FITC conjugated anti-*P. gingivalis* IgG-labeled, green) were passed through. *P. gingivalis-S. gordonii* microcolonies (yellow) were visualized by confocal microscopy. The number of dual species microcolonies formed was significantly greater in the CSE-exposed cells compared to the controls (*p* < 0.01). Observation first published in [31] but data previously unpublished in this form. **(E)** A model of cigarette smoke extract-induced alterations to the surface of the periodontal pathogen, *P. gingivalis*, is presented. (1) Surface expression of the major fimbrial protein (FimA), but not the minor fimbrial antigen (Mfa1), is upregulated [6]. (2) At the same time, the highly pro-inflammatory capsule is inhibited by CSE, increasing fimbrial protein bioavailability. (3) Multiple outer membrane proteins are upregulated upon cigarette smoke exposure, including the highly antigenic RagB protein [6]. The biological significance of CSE-induced alterations to the membrane proteome is currently under investigation. (4) While we have not examined *P. gingivalis* directly, the LPS profile in the saliva of smokers, compared to that of non-smokers, exhibits altered 3-OH fatty acid content derived from overall oral microbiome [55].

Increased prevalence of *P. gingivalis*, the laboratory workhorse of periodontal pathogens, has been repeatedly shown in smokers [47-50]. Furthermore, *P. gingivalis* is found in significantly higher numbers in smokers than non-smokers and infection is more persistent in smokers compared to non-smokers [41,51]. With this in mind, we have examined the mechanisms by which tobacco smoke augments *P. gingivalis* biofilm formation. These are summarized in Figure 2. CSE significantly influences the expression of 6.8% of the *P. gingivalis* genome, as determined by whole genome arrays, with several genes in the *P. gingivalis* capsular operon significantly suppressed [6]. Transmission electron microscopy confirmed that CSE exposure suppresses capsule formation in *P. gingivalis* at the ultrastructural level (Figure 2A and B). Nicotine alone has also been reported to influence extracellular polysaccharide production in *S. mutans* and *S. sanguinis*, partly explaining enhanced interactions between these two bacteria [8]. Furthermore, CSE promotes *P. gingivalis*-*Streptococcus gordonii* dual species biofilm formation [52], as shown in Figure 2C and D. Surface expression of the major fimbrial protein (FimA), but not the minor fimbrial antigen (Mfa1), is upregulated by CSE [6]. This promotes interactions with the early dental plaque colonizer, *S. gordonii*, enhancing biofilm formation [31]. Indeed, FimA mutants do not form biofilms [53]. Increased FimA production may also aid *P. gingivalis* survival by suppressing the TLR-mediated inflammatory response to this pathogen [31]. As noted earlier, the highly pro-inflammatory capsule is inhibited by CSE, which increases fimbrial protein bioavailability. Multiple outer membrane proteins are upregulated upon cigarette smoke exposure, including the highly antigenic RagB protein [6]. The biological significance of CSE-induced alterations to the membrane proteome is currently under investigation. *P. gingivalis* produces several types of lipopolysaccharide (LPS) that comprise the external leaflet of the Gram-negative outer membrane. The inflammatory potential of these LPS isoforms is highly variable. Penta-acylated lipid A isoforms efficiently induce inflammation upon engagement of TLR4 on innate immune cells while tetra-acylated lipid A isoforms are antagonistic [54]. In addition to acylation, the length of lipid A 3-OH fatty acid chains also influences inflammatory capacity. While we have not examined *P. gingivalis* individually, lipid A-derived 3-OH fatty acid profiles in the saliva of smokers are altered, compared to that of non-smokers, and are consistent with an oral biofilm of reduced inflammatory potential [55]. A model of CSE-induced alterations to *P. gingivalis* physiology is presented in Figure 2E.

## Conclusions

Since the discovery in 2010 that cigarette smoke extract augments biofilm formation in the oral pathogen, *P. gingivalis* [52], it has become apparent that smoking promotes bacterial adhesion and biofilm formation in several other key pathogens, including *S. mutans*, *S. aureus*, *P. aeruginosa* and *S. pneumoniae*. Enhanced bacterial evolution, including the emergence of antibiotic resistance, protection from antibiotics and other antimicrobials, immune response shielding and the increased potential for secondary colonization each have clear implications to disease treatment for the present and the future. However, it is clear that when it comes to understanding the underlying mechanisms we are currently just “scratching the surface”.
